# Inert Gas Mild Pressure Action on Healthy Humans: The “IPA” Study

**DOI:** 10.3390/ijms252212067

**Published:** 2024-11-10

**Authors:** Costantino Balestra, Clément Leveque, Simona Mrakic-Sposta, Mathias Coulon, Romain Tumbarello, Alessandra Vezzoli, Gerardo Bosco, Zuha Imtiyaz, Stephen R. Thom

**Affiliations:** 1 Environmental, Occupational, Aging (Integrative) Physiology Laboratory, Haute Ecole Bruxelles-Brabant (HE2B), 1160 Brussels, Belgium; clement.leveque.kinepro@gmail.com (C.L.); mcoulon@he2b.be (M.C.); rtumbarello@he2b.be (R.T.); 2 Anatomical Research and Clinical Studies, Vrije Universiteit Brussels (VUB), 1090 Brussels, Belgium; 3 DAN Europe Research Division (Roseto-Brussels), 1160 Brussels, Belgium; 4 Physical Activity Teaching Unit, Motor Sciences Department, Université Libre de Bruxelles (ULB), 1050 Brussels, Belgium; 5 Institute of Clinical Physiology-National Research Council (CNR-IFC), 20142 Milano, Italy; simona.mrakicsposta@cnr.it (S.M.-S.); alessandra.vezzoli@cnr.it (A.V.); 6 Department of Biomedical Sciences, University of Padova, 35131 Padova, Italy; gerardo.bosco@unipd.it; 7 Department of Emergency Medicine, School of Medicine, University of Maryland, Baltimore, MD 21250, USA; zimtiyaz@som.umaryland.edu (Z.I.); sthom@som.umaryland.edu (S.R.T.)

**Keywords:** extracellular vesicles, exosomes, filamentous actin, decompression sickness, diving, inert gas, interleukin-1β, microglia, microparticles, plasma gelsolin, oxidative stress, oxyinflammation, ROS, interleukin-6

## Abstract

The goal of this study was to evaluate inflammatory and oxidative stress responses in human subjects (9 females and 15 males) (age [29.6 ± 11.5 years old (mean ± SD)], height [172.0 ± 10.05 cm], and weight [67.8 ± 12.4 kg]) exposed to 1.45 ATA of helium (He) or nitrogen (N_2_) without concurrent hyperoxia. We hypothesized that elevated gas pressures would elicit an inflammatory response concurrent with oxidative stress. Consistent with ex vivo studies, both gasses elicited neutrophil activation, small elevations in microparticles (MPs) and increases in intra-MP interleukin (IL)-1β and inflammatory nitric oxide synthase, and an increase in urinary IL-6 concurrent with a marked reduction in plasma gelsolin. Mixed responses indictive of oxidative stress, with some biomarker elevations but little change in others and a decrease in some, were observed. Overall, these results demonstrate that exposure to typical diving gasses at a mildly elevated partial pressure will initiate inflammatory responses, which may play a significant role in decompression sickness (DCS). The complex pattern of oxidative stress responses may be indicative of competing systemic reactions and sampling different body fluids.

## 1. Introduction

Scuba diving is a common and relatively safe activity performed all around the world. Decompression sickness (DCS) is one of the related dangers that takes place after a rapid decrease in ambient pressure [1,2]. Decompression sickness symptoms are experienced upon ascending from underwater dives but also in other environmental changes, such as high-altitude flying and spacecraft extravehicular activity [3,4]. While rarely fatal, symptoms include central nervous system involvement and vestibular, pulmonary, tegmental [5], and muscoloskeletal disorders [6,7,8].

The occurrence of DCS is traditionally attributed to the accumulation of inert gas in the tissues while breathing at depth (higher partial pressure) and its inadequate release within safe decompression limits to form gas bubbles [9,10,11].

Vascular gas emboli (VGE) are commonly encountered after diving without eliciting symptoms. These originate from supersaturated tissue and pre-existing micronuclei that permit bubbles to be developed and whose population can change, thus altering the propensity for bubble formation [12,13,14,15].

VGE detected by ultrasound are inconsistently present in humans suffering from DCS. This has led to investigations that have added an inflammatory component to DCS pathophysiology and to the nucleation of VGE. The development of “second-generation micronuclei” has been proposed to be responsible for VGE [16,17,18]. A body of work implicates a subset of extracellular vesicles (EVs) with 0.1 to 1 µm microparticles (MPs) that are elevated in humans and rodent models exposed to high gas pressure that rises further after decompression, and some contain a gas phase that could serve as gas cavitation nuclei for gas diffusing from supersaturated tissues [19,20,21,22,23,24,25,26,27,28]. MPs initiate a systemic inflammatory response related to neutrophil activation [26,29,30,31]. Inflammatory MP production is an oxidative stress response triggered by gasses associated with diving, and it is higher with nitrogen compared to helium [32,33,34], the two inert gasses most commonly breathed [35].

While both in vitro and in vivo models have been used to assess cellular responses to pressure changes and bubble nucleation, they fail to account for the role of dissolved gasses in a physiologically relevant manner, and they have never been used at “mild” or nominal pressures where inflammatory events have been documented [36,37]. In our previous work, we analyzed oxidative stress responses while breathing oxygen and during deep diving [16,17,38]. In the current study, we wanted to further investigate the pressure and inert gas effects on oxidative stress and MP production at a mild pressure exposure of 1.45 ATA and under normoxic conditions following a profile previously investigated by our group [39,40,41].

This study couples assessments of MPs and neutrophil activation with evaluations of other biomarkers since it is well accepted that breathing oxygen at a hyperbaric pressure can lead to an increase in reactive oxygen species (ROS) [39,42]. This can result in the conditions of oxidative stress and “oxyinflammation” [43,44,45]. An excess of free radical generation that exceeds antioxidant defenses may damage biological macromolecules, including lipids, proteins, and nucleic acids. The accumulation of oxidative damage can alter health status [46], compromising immune system efficacy and inducing an inflammatory response [47].

For example, the release of 8-isoprostanes detected in scuba divers [48] is derived from the ROS-catalyzed peroxidation of unsaturated fatty acids and is used as an index of lipid peroxidation [49,50]. ROS-mediated damage to DNA is measurable as 8-oxo-7,8-dihydro-deoxyguanosine (8-OH-dG) and was detected to be increased after short hyperbaric oxygen exposure in divers that performed dives in both warm [48] and cold water [51]. In addition to ROS, an increase in nitric oxide (NO), a signaling molecule involved in the response to a hyperbaric environment [52], can occur during diving, as reported by some authors that observed remarkable increases in the plasma NO derivatives nitrate and nitrite (NOx) during a dive at a 40 m depth [53].

It is well known that scuba divers can activate endogenous antioxidant defenses [43,53,54,55] that can protect biological macromolecules from excessive antioxidant depletion and oxidative stress during a dive. However, a consequence of oxidative dysbaric stress is increased circulating inflammatory cytokines, including interleukins (IL-1β and IL-6), which activate the inflammation cascade [37,38,56]. ROS can also stimulate the expression of several inflammation-related genes of IL-6 [57]. For this reason, changes in pro-inflammatory factors, including tumor necrosis factor-alpha (TNF-α) [58], IL-1β [38], and IL-6, are expected to occur during the progression of dysbaric stress to overt decompression sickness (DCS) [59]. One additional biomarker of oxidative stress is neopterin, [60,61,62] which reflects an increase in purine metabolism that can activate cell-mediated immunity [63].

The goal of this work was to improve our understanding of cellular responses to the hyperbaric environment by separating the increased partial pressure of oxygen from the partial pressure of the so-called “inert gas”. Although many studies on oxygen levels and oxidative stress have been published from several authors, little is known to appreciate to what extend inert gasses may be part of this process, especially at moderate pressure levels [64]. Given that we know that elevated pressures of nitrogen and gasses such as helium and argon activate leukocytes via an oxidative stress process that triggers MP production [35,65], we investigated inflammatory and oxidative stress responses of mild-pressure exposure to 1.45 ATA nitrogen or helium while controlling oxygen partial pressure to just 0.21 ATA in healthy volunteers.

## 2. Results

### 2.1. Neutrophil Activation Elicited by Inert Gas

**Figure 1:**
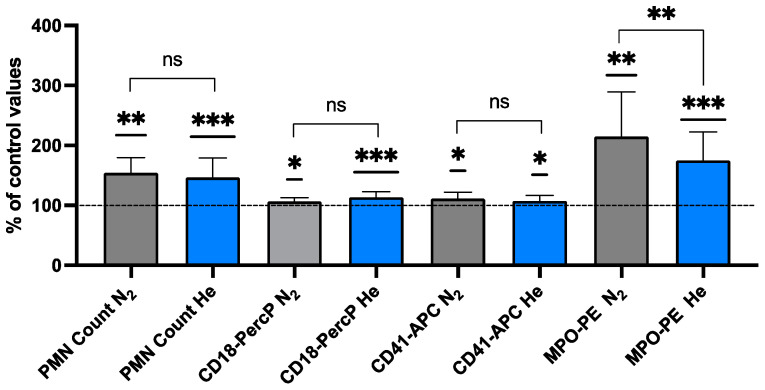
Activation of neutrophils after inert gas exposure (N_2_ grey, He Blue). Data are shown as mean ± SD expressed in % of pre-exposure values of neutrophils (identified in flow cytometer based on CD66b expression) expressing myeloperoxidase (MPO) and CD18 above threshold value as index of cell activation (one-sample *t*-test and unpaired *t*-test). (* *p* < 0.05; ** *p* < 0.01; *** *p* < 0.001; ns = Non-Significant.)

The activation of neutrophils, which express the CD66b protein, was assessed by flow cytometry as a surface expression of the CD18 protein component of the β_2_ integrin and myeloperoxidase (MPO, a manifestation of degranulation) against the background of CD66b-positive cells (Table 1 and Figure 1).

We also assessed the presence of CD41, a platelet-specific protein, on the neutrophil surface. Studies have shown that platelets shed MPs upon activation and that platelets and their MPs can adhere to neutrophils [26,66,67]. An assessment for the presence of pGSN on the neutrophil surface was motivated by recent published observations showing a decrease in pGSN in blood and on neutrophils associated with high-pressure exposure. We found that every measured parameter of neutrophil activation shows a significant change, but the only statistically significant difference between helium and nitrogen exposure was found in myeloperoxidase expression. Nitrogen was more stimulating at 214.7 + 74.8% compared to 175.0 + 47.6% for helium.

### 2.2. Blood-Borne EVs Elicited by Pressurized Inert Gas Exposure

**Figure 2:**
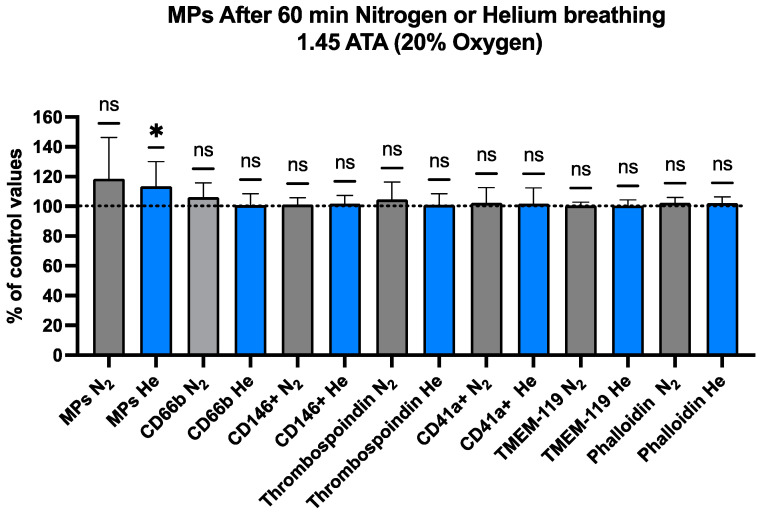
Microparticles in blood after helium or nitrogen exposure (N_2_ grey, He Blue). Flow cytometry was used to evaluate MPs. Relative variations expressed in % of pre-exposure value of each that expressed proteins specific to different cells, including neutrophils (CD66b), endothelial cells (CD146), platelets (CD41a), and microglia (transmembrane protein 119, TMEM). As discussed in text, proteins expressing TSP-1 and F-actin, evaluated as those binding phalloidin, were also assessed. Data are shown as mean ± SD (* = *p* < 0.05; ns = non-significant; *t*-test vs. control, with everyone acting as its own control (one-sample *t*-test)).

Blood counts are shown for MPs in Figure 2 and Table 2. MPs were identified based on the size (0.1–1 µm) and surface expression of annexin V (which binds to phosphatidylserine). In addition to the total number of particles, the expression of surface proteins on MPs originated from neutrophils (CD66b), platelets (CD41), endothelial cells (CD146), and microglia (TMEM119) was analyzed. To gain further insights into MPs, the surface expressions of thrombospondin-1 (TSP) and filamentous (F-) actin (staining with phalloidin) were also assessed. Recent work in the murine model has indicated that F-actin and TSP-expressing MPs have an inflammatory role [65]. Both helium and nitrogen exposure showed increases, but the only one that reached statistical significance was the total MP count for helium exposure (113.3 ± 16.7%).

### 2.3. IL-1β, iNOS, and Gelsolin Changes

**Figure 3:**
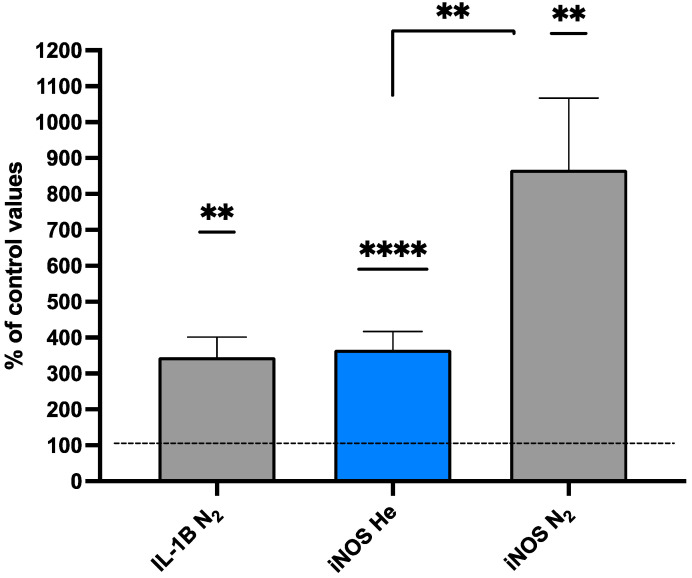
IL-1B and iNOS (% of control values) (N_2_ grey, He Blue). Data are shown as mean ± SD (** *p* < 0.01; **** *p* < 0.0001; ns = not significant; Wilcoxon and Mann–Whitney tests).

IL-1β secretion requires unconventional pathways involving packaging into either MPs or exosomes to be liberated to the extracellular milieu [68]. With high-pressure exposure, virtually all IL-1β are found within MPs [38]. The intra-MP IL-1β values post-pressure exposure are shown in the first two columns of Figure 3 and expressed as the % change from baseline. Due to a technical error, pre-exposure values were only obtained for the group exposed to nitrogen (*n* = 9) and found to be 11.6 + 6.1 pg/million MPs. The post-helium group IL-1β value was 33.7 + 47.2, whereas that for the nitrogen group was 38.1 + 32.9 pg/million MPs. As shown in Figure 3, the post-exposure values for both groups were statistically significantly different from the baseline, but the values were not significantly different between the gas exposures.

Animal, human, and cells studies suggest that there is a role for iNOS in immunoregulatory function and inflammatory responses [69]. Animal studies suggest that there is a role for iNOS in generating a gas phase within MPs, and iNOS elevations have also been shown to have a role in a study of open-water scuba divers [29,38]. The values for intra-MPs iNOS are shown in Table 3. The baseline iNOS values between the helium and nitrogen groups are significantly different (*p* < 0.001). The value shown for the nitrogen group is virtually the same as reported for other groups of divers as a baseline [38] (Arya, 2023). There is no explanation for the markedly high value seen in the baseline helium group. Despite the baseline variability, the post-exposure iNOS values were significantly different in both the helium and nitrogen groups (Table 3; Figure 3).

#### Plasma Gelsolin (pGSN) After Inert Gas Exposure

**Figure 4:**
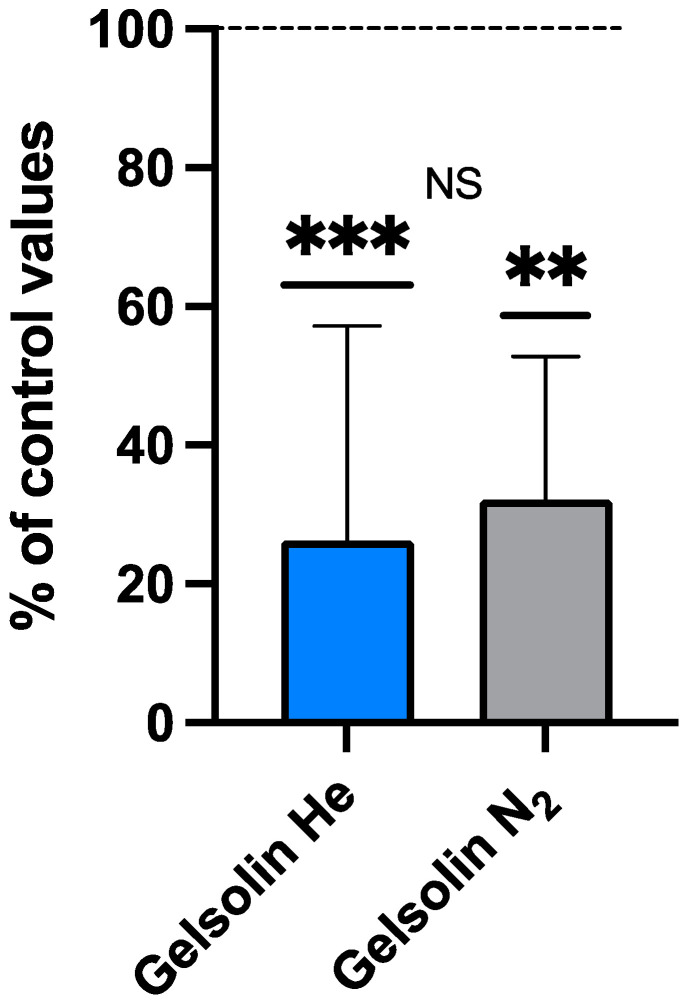
Plasma gelsolin (% of control values) (N_2_ grey, He Blue). Data are shown as mean ± SD (** *p* < 0.01; *** *p* < 0.001; Wilcoxon and Mann–Whitney tests; NS = not significant).

Plasma gelsolin is a highly conserved actin-binding protein that has been reported to decrease in post-exposure samples in a murine DCS model and human subjects exposed to pressure in a hyperbaric chamber [38]. The comparison between the same exposure to different breathed inert gasses shows a significant reduction in pGSN after either helium or nitrogen exposure and no significant difference between the gas responses (Table 3; Figure 4).

### 2.4. Oxyinflammation After Inert Gas Exposure

**Figure 5:**
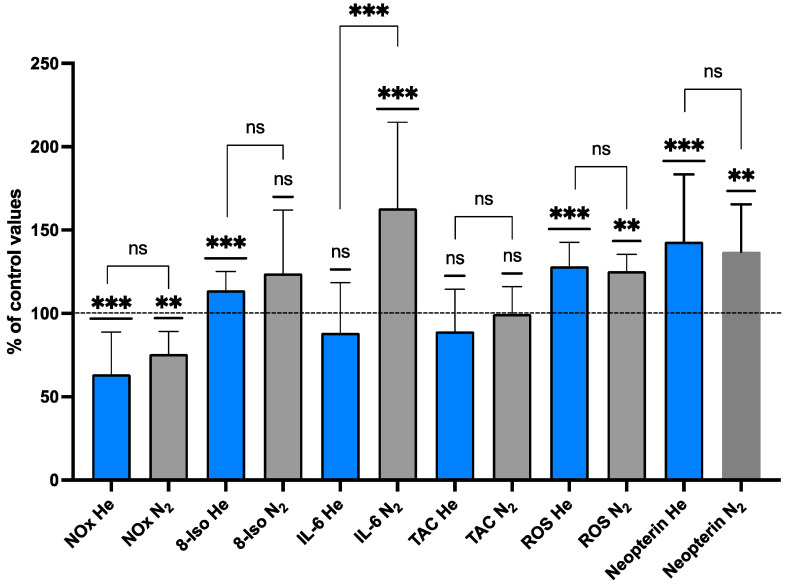
Oxyinflammation % of control values (N_2_ grey, He Blue)**.** Data are shown as mean ± SD. ** *p* < 0.01; *** *p* < 0.001; ns = not significant; *t*-test and one-sample *t*-test.

The term “oxyinflammation” has been coined for the combination of systemic oxidative stress associated with an inflammatory condition, and it can be seen in association with conditions of hyperoxia and in high-pressure activities. To assess oxidative stress, we measured the ROS production rate and Total Antioxidant Capacity in saliva samples, and urine was probed for 8-iso-PGF2α (a biomarker of lipid peroxidation), neopterin, NO metabolites, and IL-6 in subjects pre- and post-inert gas exposure (N_2_ vs. He). The data are displayed in Figure 5 and Table 4. Several changes with statistically significant differences in the ROS production level (μmol⋅min^−1^) and interleukin 6 (pg·mL^−1^) were found between pre- and post-exposure in the N_2_ groups. Furthermore, a significant difference was observed in the ROS production level (μmol⋅min^−1^), neopterin (mM⋅mol^−1^creatinine), and NOx (µM) after He exposure (Table 4).

**Figure 6:**
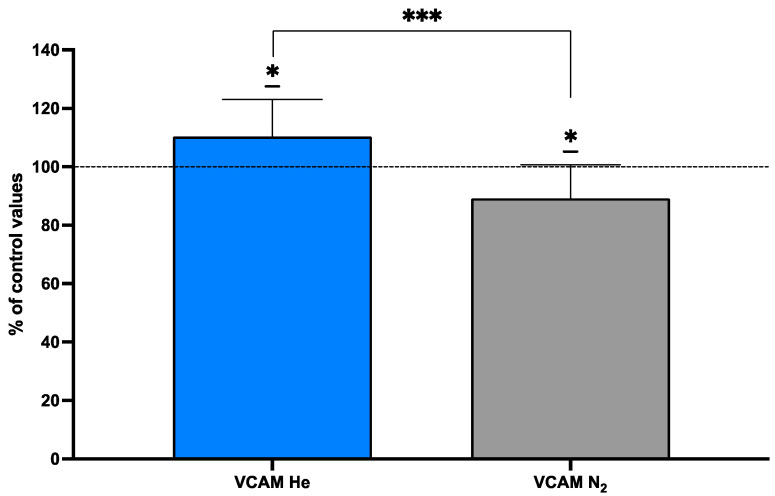
Endothelial markers (N_2_ grey, He Blue). VCAM-1. Data are shown as mean ± SD. * *p* < 0.05; *** *p* < 0.001; *t*-test and one-sample *t*-test.

We did not find any statistically significant differences in the TAC (mM) in either group (Figure 5). Interleukin 6 in urine showed a significant increase after N_2_ exposure and a mild decrease after He exposure, resulting in a highly significant difference for interleukin 6 production between N_2_ and He after exposure (*p* < 0.0001) (Figure 6).

### 2.5. Endothelial Markers After Inert Gas Exposure

Vascular cell adhesion protein 1 (VCAM-1) functions as a cell adhesion molecule. VCAM-1 was assessed in subjects pre- and post-inert gas exposure (N_2_ vs. He) (Table 5), and the results are displayed in Figure 6. Following gas exposure, the blood-borne VCAM-1 value was increased to 110.4 ± 12.6% for helium but decreased to 89.25 ± 11.5% for nitrogen, although absolute values were not significantly different.

## 3. Discussion

This study was conducted to advance our understanding of oxyinflammation as a diving response by focusing on the effects of He and N_2_ without concurrent hyperoxia. Ex vivo studies with neutrophils have demonstrated that gasses associated with diving, namely He, N_2_, and Ar, can initiate an oxidative stress response by triggering the production of singlet oxygen with the subsequent activation of iNOS and NADPH oxidase [35]. Elevations in oxygen tension associated with breathing air at elevated pressures as high as 790 kPa were shown to have no significant impact on MP production and the associated responses in a murine model, but no similar studies have been conducted with human subjects [70]. The pressure threshold for gasses triggering the singlet oxygen-mediated responses in neutrophils ex vivo was found to be ~186 kPa [35]. We chose to carry out the current trial at a slightly lower pressure of 145 kPa.

Neutrophil activation occurred with both He and N_2_ exposure. The production of MPs was nominal, which we attribute to the use of gas partial pressures slightly below the threshold found for neutrophils ex vivo. Elevations in IL-1β and IL-6 following high gas pressure exposure has been described in a murine model, in cells, and in humans after simulated diving [28,71]. Urinary levels of IL-6 can mirror plasmatic levels as already reported [72,73,74]. Oxidative stress triggered by He and N_2_ activate the NLRP3 inflammasome concurrent with MP production [35]. This element of gas-mediated neutrophil activation was observed in the current study as the elevation of intra-MP IL-1β. The increase in iNOS within MPs is consistent with the same series of events and has been observed in other recent studies [38]. There is a difference in potency between He and N_2_ in ex vivo neutrophil activation studies. We found a significant difference between He and N_2_ for MP iNOS cargo and plasma IL-6, but no significant difference was found in elevations in intra-MP IL-1β in our current investigation.

Plasma gelsolin (pGSN) blood levels fall in numerous acute and chronic inflammatory states. Among studies, the magnitude of pGSN reduction parallels the extent of tissue damage, and depletion precedes and predicts adverse clinical outcomes [75,76,77,78,79,80,81]. In the present study, we observed significantly lower pGSN concentrations in post-dive plasma. We view pGSN as a physiological antagonist to MP-mediated inflammation. Prior work suggests that the decrease occurs due to pGSN consumption from the binding and lysis of pro-inflammatory F-actin-expressing MPs [36,82]. We have observed pGSN reduction post-diving in human and murine studies [36,37,38]. In a murine DCS model and numerous animal studies of infection, injury, and inflammation, pGSN supplementation can abrogate organ damage [83].

With regard to oxidative stress responses, we observed a complex pattern of responses between He and N_2_. This may be due to competing reactions with this systemic exposure (versus more simple studies with ex vivo neutrophils) and responses of multiple organs and tissues, as well as sampling from multiple body fluids. Our data show an increase in ROS production levels in saliva without an increased oxygen partial pressure (N2: +28%; He: +25%) and, consequently, lipid peroxidation (8-iso-PGF2) in urine samples (N2: +13%; He: +15%) with both breathed gasses. However, no significant changes were observed for salivary TAC, urinary Nox, or neopterin.

VCAM-1 is expressed by the vascular system and can be induced by ROS, IL-1β, or TNFα, which are produced by many cell types. Endothelial activation with elevations in plasma VCAM-1 were reported following high-pressure exposure in several studies [84,85,86]. In our study, the levels of VCAM-1 followed different trajectories with an increase after helium exposure and a decrease after nitrogen exposure. This seeming contradiction may reflect subtle differences in oxidative stress and compensatory responses. That is, He exposure triggered a significant increase in plasma MPs, including those carrying IL-1β. These MPs cause vascular damage in the murine model. Nitrogen-induced MPs with their significantly higher iNOS content may have altered the balance of responses given the potent vasodilatory effects of NO.

There is no precedence [64,87,88,89,90] for cell activation or immune responses related to hydrostatic pressure elevations as used in this study [91]. However, elevated partial pressures of inert gasses (in this case, He and N2) can cause oxidative stress, leading to immune responses including microparticle formation that result from oxygen–inert gas collision complexes to yield reactive singlet oxygen [35,92].

In conclusion, inert gas exposure at 1.45 ATA resulted in biomarker concentration changes. Based on these findings, new studies must focus on exposure times, depth, and biological sample collection times and investigate physiological stress responses. There are also limitations to our study that need to be recognized. As is typical of these human subject projects, the relatively small sample size poses limits on generalizations. There are also variable effects observed in the baseline values for iNOS between the He and N_2_ groups that are unexplained. Diurnal variations may also count, but to limit this interference, our experiments were conducted between 8 and 12:00 h during weekends.

## 4. Materials and Methods

### 4.1. Experimental Protocol

A total of 24 healthy subjects (9 females and 15 males) volunteered for this study after approval from the Bio-Ethical Committee for Research and Higher Education, Brussels (N° B200-2020-088), and written informed consent was obtained. All experimental procedures were conducted in accordance with the Declaration of Helsinki [93]. Analyses of deidentified blood samples were approved by the University of Maryland Institutional Review Board (N^o^ HP-00059996).

**Figure 7:**
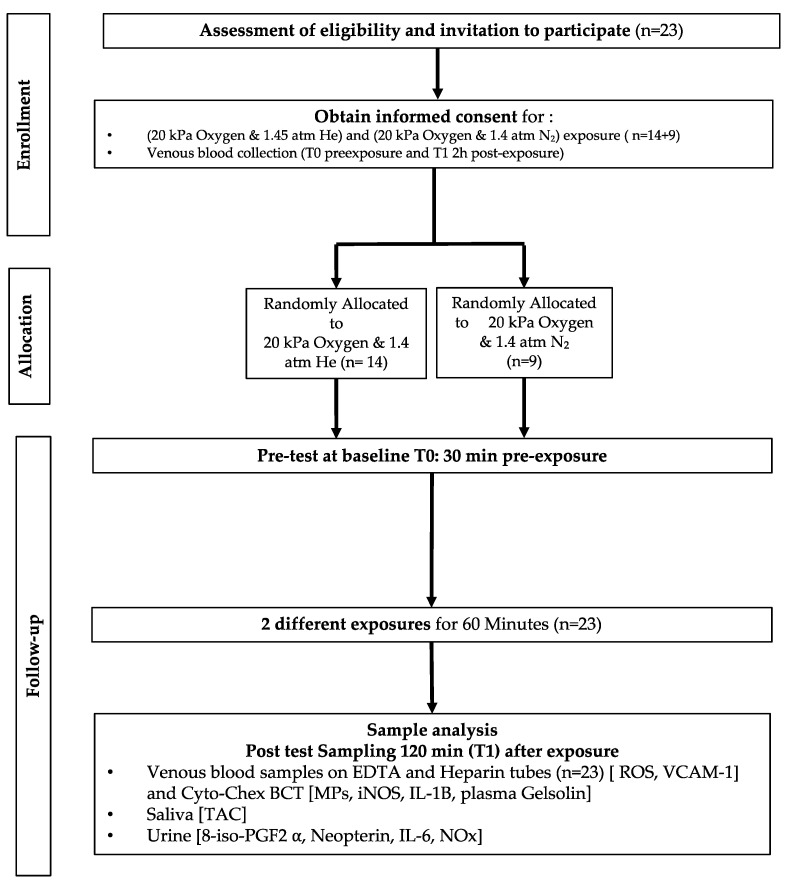
Experimental flowchart.

After medical screening to exclude any comorbidity, participants were prospectively randomized into 2 groups to receive either a breathing mix with helium or nitrogen under pressure at 1.45 ATA with oxygen at 14.5% to reach roughly 0.21 of the inspired fraction of oxygen to mimic the atmospheric air oxygen content. (Figure 7). As far as age [29.6 ± 11.5 years old (mean ± SD)], height [172.0 ± 10.05 cm], weight [67.8 ± 12.4 kg], gender ratio, and health status are considered, the groups were comparable.

Inert gas exposure was carried out for 1 h by means of an orofacial “full-face” mask (Ocean Reef, Genoa, Italy) attached to the mixture tank, with care being taken to fit and tighten the mask on the subject’s face. Participants were pressurized in one Revitalair technology (1.45 ATA) (Biobarica Revitalair 430, Buenos Aires, Argentina) hyperbaric monoplace chamber.

Blood, saliva, and urine samples were obtained before exposure (T0) and 2 h after the end of inert gas administration.

### 4.2. Experimental Protocol for Microparticle Analysis

The blood (~5 mL) was drawn into Cyto-Chex BCT test tubes that contained a proprietary preservative (Streck, Inc., Omaha, NE, USA). The blood was centrifuged for 5 min at 1500× *g*, and the supernatant was 12.5 mmol/L EDTA to impede MP aggregation and then centrifuged at 15,000× *g* for 30 min. Aliquots of the 15,000× *g* supernatant were stained with antibodies for MP analysis by flow cytometry, and a portion was used for exosome analysis. Samples were sent by express mail to the University of Maryland (Dr. Thom) and to the IFC-CNR Milano, Italy (Dr. Mrakic-Sposta) laboratory, where all analyses were performed following published techniques described in previous publications [25,43]. In brief, total MPs and subtypes were assayed in an 8-color, triple-laser MACSQuant (Version 2.13.3, Miltenyi Biotec Corp., Auburn, CA, USA) flow cytometer with the manufacturers’ acquisition software using standard methods, including a “fluorescence minus one control test”. This analysis provides a way to define the boundary between positive and negative particles in an unbiased manner by defining the maximum fluorescence expected for a given subset after outlining the area in a two-dimensional scatter diagram when a fluorophore-tagged antibody is omitted from the stain set. The analysis allows for a simple decision as to where to place the upper boundary for non-staining particles in a fluorescence channel. All supplies, reagents, and manufacturer sources have been described in previous publications [94,95,96]. The blood was centrifuged for 5 min at 1500× *g*, and the supernatant was 12.5 mmol/L EDTA to impede MP aggregation and then centrifuged at 15,000× *g* for 30 min. Aliquots of the 15,000× *g* supernatant were stained with antibodies for MP analysis by flow cytometry, and a portion was used for exosome analysis. Plasma stored at −80 °C after a 15,000× *g* centrifugation step preceding an MP analysis was used for IL-1, NOS2, and pGSN assays.

### 4.3. Reagents (Microparticle Analysis)

Chemicals were purchased from Sigma-Aldrich (St. Louis, MO, USA) unless otherwise noted. Annexin binding buffer and the following agents were purchased from BD Pharmingen (San Jose, CA, USA): fluorescein isothiocyanate (FITC)-conjugated Annexin V (cat# 556419), R-PE-conjugated anti-human CD18 (cat#555924), and PerCP/Cy5.5-conjugated anti-human CD41a (cat #340931). APC-conjugated anti-human CD146 (cat #340931) was purchased from BioScience (San Diego, CA, USA), AlexaFluor488-conjugated anti-human TMEM119 (cat #FAB103131G) was from R & D Systems (Minneapolis, MN, USA), anti-thrombospondin (TSP)-1 (cat #sc-393504) was from Santa Cruz Biotechnology (Dallas, TX, USA), and FITC-conjugated anti-human myeloperoxidase (MPO, cat# HM1051PE-100) was from Hycult Biotech (Plymouth Meeting, PA, USA). Antibodies purchased from Biolegend (San Diego, CA, USA) included AlexaFluor647-conjugated anti-human CD63 (cat #353016), PercpCy5.5-conjugated anti-human CD81 (cat #349520), and BV421-conjugated anti-human CD66b (cat#347201).

### 4.4. Neutrophil Activation Analysis

Whole fixed blood from the Cyto-Chex tubes (100 µL) was stained for 30 min at room temperature in the dark with optimized concentrations of antibodies as listed above. After staining, 2 mL of phosphate-buffered saline (PBS) was added to dilute each sample tube prior to analysis, with the cytometer acquisition set to use anti-human CD66b as the fluorescence trigger to recognize neutrophils.

### 4.5. IL-1β and NOS2 Measurements

Human-specific ELISA Kits (eBioscience, San Diego, CA, USA) that detect pro- and mature forms of IL-1β or NOS2 were used following the manufacturer’s instructions. Measurements were performed using plasma supernatant after blood was centrifuged at 15,000× *g*, as described for flow cytometry studies, and also in supernatant and pellet fractions separated by a second centrifugation at 21,000× *g* for 30 min. The MPs in pellets were placed in 0.3 mL lysis buffer, the protein content of the sample was measured and diluted to 5 mg/mL, and 20 µg of protein was used for analysis.

### 4.6. Gelsolin Assay

A human-specific commercial pGSN ELISA kit (LSBio, Inc., Seattle, WA, USA) was used following the manufacturer’s instructions. Serial dilutions in PBS were prepared using the supernatant after 15,000× *g* centrifugation of plasma as described above and analyzed concurrently with a range of known pGSN standards.

### 4.7. Exosome/Lipophilic Particles Assay

Using 15,000× *g* supernatants from plasma, as described above, 5 µL of samples was diluted in 100 µL of PBS and incubated with dyes [2.5 µmol PKH67 (Sigma Cat#SIG-MINI67), 2.5 µmol Laurdan (1-[6-(Dimethylamino)-2-naphthalenyl]-1-dodecanone, Tocris Biotech Cat#7275)], and antibodies for 30 min prior to analysis using an ImageStream^®^X Mk II: Imaging Flow cytometer. FluoSphere™ carboxylate-modified microspheres (Thermofisher, 20 and 100 nm in diameter) were used to provide size bracketing, and initial standardization of methods was conducted with 120 nm (range 80–140 nm) synthetic lipid vesicles from Cellarcus Biosciences (San Diego, CA, USA) that were made with a lipid composition comparable to mammalian cells. For initialization, the bright field instrument and lasers were set to maximum power, side scatter (SSC) was set to 70 mW, and the 60X imager magnification was set to high gain. Channel 1 was used for bright field, and Ch.6 for SSC. After setting the compensation matrix with the bright field off and all channels enabled, single-color compensations were set for each color, with gates set to detect particles between 20 and 100 nm in diameter.

### 4.8. Blood Collection for Other Biomarkers

Each blood sample for other plasmatic measures consisted of approximately 15 mL of venous human blood collected in lithium heparin and EDTA tubes (Vacutainer, BD Diagnostic, Becton Dickinson, Italia S.p.A., Milan, Italy). Plasma and red blood cells (RBCs) were separated by centrifugation (Eppendorf Centrifuge 5702R, Darmstadt, Germany) at 1000× *g* at 4 °C for 10 min.

All samples collected were sent by express mail to the IFC-CNR Milano, Italy (Dr. Mrakic-Sposta) laboratory, where all analyses were performed. Samples were then stored in multiple aliquots at −80 °C until assayed and thawed; an analysis was performed within one month from collection.

#### Vascular Cell Adhesion Protein (VCAM-1)

Vascular cell adhesion protein 1 (VCAM-1) concentrations were obtained in plasma using the ELISA kit (Cat. No. EH0326, Fine Test, Wuhan, China) according to the manufacturer’s instructions. Samples were read at a wavelength of 450 nm. The concentration of VCAM-1 was calculated by drawing a standard curve. Coefficient of variation (CV) assay indicated by the manufacturer: inter-assay CV, 5.92%; intra-assay CV, 4.68%.

### 4.9. Saliva Collection and Biomarker Assessment

Approximately 1 mL of saliva was obtained using Salivette devices (Sarstedt, Nümbrecht, Germany). The subjects were instructed on the correct use (i.e., no eating, drinking, or oral intake of drugs 1 h before sample collection). Salivettes were centrifuged at 1500× *g* 20 min at 4 °C, and retrieved saliva was transferred, aliquoted, and then stored at −80 °C until assayed and thawed only once before analysis.

#### 4.9.1. Reactive Oxygen Species (ROS)

An X-band electron paramagnetic resonance spectroscopy (EPR, 9.3 GHz) (E-Scan, Bruker Co., Billerica, MA, USA) detected ROS production in saliva samples (analysis software version 2.11, Win EPR System). Methods were previously described [17]. Briefly, spin probe CMH (1-hydroxy-3-methoxy-carbonyl-2,2,5,5-tetramethylpyrrolidine) was used for ROS determination, and a stable radical CP^●^ (3-carboxy2,2,5,5-tetramethyl-1-pyrrolidi-nyloxy) was used as an external reference to convert ROS determinations into absolute quantitative values (µmol^.^min^−1^).

#### 4.9.2. Total Antioxidant Capacity (TAC)

The 6-hydroxy-2,5,7,8-tetramethylchroman-2-carboxylic acid (Trolox-) equivalent antioxidant capacity assay, a widely used kit-based commercial method (Cayman Chemical, Ann Arbor, MI, USA; Item No. 709001), was used as previously described [17]. Briefly, 10 µL of saliva was added in duplicate to 10 μL of metmyoglobin and 150 μL of the chromogen solution.

Then, reactions were initiated through the addition of 40 μL of H_2_O_2_, as indicated by the instructions. Reaction mixtures were incubated at room temperature for 3 min. Samples were read at a wavelength of 750 nm (Infinite M200, Tecan, Austria, operating in Window system). Coefficient of variation (CV) assay indicated by the manufacturer: inter-assay CV, 3%; intra-assay CV, 3.4%.

### 4.10. Urine Collection

Urine samples were collected by the participants through voluntary voiding in a sterile container. Biofluids were stored at 4 °C in a cooler and then stored in multiple aliquots at −20 °C until assayed.

#### 4.10.1. Lipid Peroxidation (8-iso-PGF2α)

Lipid peroxidation was assessed in urine using a competitive immunoassay of 8-isoprostane concentration (8-iso-PGF2α; Item No. 516360, Cayman Chemical, Ann Arbor, MI, USA). 8-iso-PGF2α concentrations were determined using a standard curve. Samples and standards were read at a wavelength of 412 nm (Infinite M200, Tecan, Austria, operating in Window system). This method has been previously described [97,98]. Coefficient of variation (CV) assay indicated by the manufacturer: inter-assay CV, 11.5%; intra-assay CV, 8.9%.

#### 4.10.2. Interleukin-6 (IL-6)

According to the manufacturer’s instructions, IL-6 urinary levels were determined using an ELISA kit (Cayman Chemical, Ann Arbor, MI, USA, Item No. 501030). This method has been previously described [17]. Coefficient of variation (CV) assay indicated by the manufacturer: inter-assay CV, 13.32%; intra-assay CV, 7.64%.

#### 4.10.3. NO Metabolites (NOx)

NO derivatives, nitrate, and nitrite (NO_2_ + NO_3_ = NOx) were measured in urine samples using a colorimetric method based on the Griess reaction (Cayman Chemical, Ann Arbor, MI, USA; Item No. 780001) as previously described [99]. Samples were read at 545 nm (Infinite M200, Tecan, Austria, operating in Window system), and a standard curve assessed the concentration. Coefficient of variation (CV) assay indicated by the manufacturer: inter-assay CV, 3.4%; intra-assay CV, 2.7%.

#### 4.10.4. Neopterin

Urinary neopterin concentrations were measured in urine using the high-performance liquid chromatography (HPLC) method [100,101]. The calibration curves were linear over the range of 0.125–1 μmol/L for neopterin levels (Varian Star chromatography workstation version 6.41). Inter-assay and intra-assay coefficients of variation were <5%.

### 4.11. Statistical Analysis

Data are presented as mean ± SD. Normality of the data was verified by means of the Shapiro–Wilk test, which allowed us to assume a Gaussian distribution. Compared to the baseline (T0—pre-exposure), data were analyzed with a paired *t*-test for intragroup comparison if Gaussian distribution was not warranted, and a Wilcoxon test was preferred. When appropriate, unpaired *t*-test or Mann–Whitney test was used for intergroup comparison. Taking the baseline measures as 100% (T0), percentage or fold changes were also calculated for each exposure protocol, allowing for an appreciation of the magnitude of change rather than the absolute values; statistical significance was then assessed by means of a one-sample *t*-test. In the figures, data are presented as percentage changes, taking each individual pre-exposure value as baseline (100%). All data are presented as mean ± standard deviation (SD); Cohen’s d with 95% CI was used to calculate the size effect. A threshold of *p* < 0.05 was considered statistically significant.

## Figures and Tables

**Table 1:** Activation of neutrophils from all participants in exposure to both inert gasses. Data are shown as mean ± SD (*n* = sample number) % of neutrophils and median fluorescence (identified in flow cytometer based on CD66b expression) expressing myeloperoxidase (MPO), CD41, plasma gelsolin, and CD18 above threshold value as index of cell activation. Pre- and post-exposure results are shown as mean ± SD. (Paired *t*-test: * *p* < 0.05, ** *p* < 0.01, and *** *p* < 0.001.) (Two post-exposure blood samples were unusable in N_2_ group.)

Exposition	Baseline	After 120 min	*p* Value	Size Effect	*n*
He 1.45 ATA (20 kPa oxygen)	MPs/μL	MPs/μL	Paired *t*-test	Cohen’s d	
% CD18	1.31 ± 0.433	3.66± 1.579	<0.001 ***	−1.581	14
Median CD18	18.44 ± 5.826	20.72 ± 6.117	<0.001 ***	−1.613	14
% CD41	3.91 ± 1.933	6.11 ± 1.860	0.002 **	−1.020	14
Median CD41	57.52 ± 8.568	61.09 ± 1.869	0.012 *	−0.779	14
% Gelsolin	5.57 ± 1.223	3.38 ± 1.321	<0.001 ***	1.460	14
Median—gelsolin	25.86 ± 2.012	23.91 ± 1.652	<0.001 ***	1.508	14
% MPO	1.40 ± 0.547	3.49 ± 1.149	<0.001 ***	−1.790	14
Median MPO	5.13 ± 1.178	5.13 ± 1.178	<0.001 ***	−1.623	14
N_2_ 1.45 ATA (20 kPa oxygen)					
% CD18-	1.43 ± 0.669	3.80 ± 1.446	0.003 **	−1.768	7
Median	17.83 ± 4.247	18.92 ± 4.309	0.020 *	−1.186	7
% CD41	4.13 ± 1.150	7.69 ± 3.037	0.009 **	−1.453	7
Median	57.87 ± 6.199	64.08 ± 7.968	0.043 *	−0.964	7
% Gelsolin	4.81 ± 1.321	2.22 ± 1.064	0.001 **	2.232	7
Median	22.20 ± 4.128	17.13 ± 3.748	0.012 *	1.331	7
% MPO	1.18 ± 0.536	3.41± 1.206	<0.001 ***	−2.281	7
Median	2.32 ± 0.752	4.82 ± 1.659	0.002 **	−1.970	7

**Table 2:** Absolute values for microparticle-derived responses in MPs/μL. Results are given as mean ± SD. (Paired *t*-test: * *p* < 0.05.)

Exposure (60 min)	Baseline	120 min Post	*p* Value	Size Effect	*n*
He 1.45 ATA (20 kPa oxygen)	MPs/μL	MPs/μL	Paired *t*-test	Cohen’s d	
Total MPs	791.91 ± 120.230	889.24 ± 148.106	0.026 *	−0.6707	14
CD66b+	9.43 ± 1.050	9.48 ± 1.158	0.775	−0.0779	14
CD146+	20.02 ± 0.689	20.35 ± 1.219	0.290	−0.2950	14
Thrombospoindin1	9.59 ± 0.832	9.68 ± 1.137	0.642	−0.1272	14
CD41a+	7.08 ± 1.552	7.16 ± 1.599	0.703	−0.1042	14
TMEM-119	29.67 ± 0.712	29.78 ± 1.032	0.705	−0.1035	14
Phalloidin	17.51 ± 0.567	17.85 ± 0.815	0.104	−0.4673	14
N_2_ 1.45 ATA (20 kPa oxygen)					
Total MPs	742.13 ± 188.954	844.04± 141.385	0.115	−0.589	9
CD66b+	9.63± 0.736	10.15 ± 0.431	0.111	−0.597	9
CD146+	21.40 ± 1.533	21.57 ± 0.948	0.611	−0.176	9
Thrombospoindin1	8.81 ± 0.602	9.16 ± 0.530	0.295	−0.374	9
CD41a+	5.70 ± 0.653	5.78 ± 0.411	0.685	−0.140	9
TMEM-119	30.07 ± 0.697	30.22 ± 0.317	0.535	−0.216	9
Phalloidin	17.85 ± 0.932	18.21 ± 0.618	0.148	−0.534	9

**Table 3:** Intra-MPs, iNOS (pg/million MPs), and plasma gelsolin μg/mL. Data are shown as mean ± SD (n = sample number) pre- and post-exposure for both inert gasses. Results are shown as mean ± SD. (Wilcoxon test: ** *p* < 0.01, and *** *p* < 0.001.)

Exposure (60 min)	Baseline	Post-120 min of Exposure	*p* Value	Size Effect	*n*
He 1.45 ATA (20 kPa oxygen)	Pre	Post	Wilcoxon	Cohen’s d	
IL-1β (pg/million MPs)	-	33.7 ± 47.2	-	-	13
iNOS (pg/million MPs)	0.174 ± 0.0952	0.510 ± 0.258	<0.001 ***	−1.00	14
Gelsolin (μg/mL)	190 ± 226.5	26.0 ± 22.3	0.931	−0.0245	14
N_2_ 1.45 ATA (20 kPa oxygen)					
IL-1β (pg/million MPs)	11.6 ± 6.14	38.1 ± 32.9	0.004 **	−1.00	9
iNOS (pg/million MPs)	0.0503 ± 0.033	0.347 ± 0.126	0.001 **	−1.00	9
Gelsolin (μg/mL)	147.7 ± 56.9	42.7 ± 26.9	0.004 **	1.36	9

**Table 4:** ROS production rate (mmol·min^−1^) and TAC (mM) in saliva, lipid peroxidation (pg⋅mol^−1^creatinine), neopterin, IL 6 (pg·min^−1^), and NOx (mM) in urine. Data are shown as mean ± SD (n = sample number) pre- and post-exposure for both inert gasses (paired *t*-test: * *p* < 0.05; ** *p* < 0.01; *** *p* < 0.001).

Exposure (60 min)	Baseline	Post-120 min of Exposure	*p* Value	Size Effect	*n*
He 1.45 ATA (20 kPa oxygen)	Value	Value	Paired-t test	Cohen’s d	
ROS—He (μmol⋅min^−1^)	0.240 ± 0.011	0.308 ± 0.009	<0.001 **	−4.778	14
TAC—He (mM)	0.244 ± 0.061	0.238 ± 0.045	0.131	0.640	14
8-iso-PGF2 α -He (pg⋅mg^−1^ creatinine)	224.3 ± 74.90	258.4 ± 61.63	0.190	−0.552	14
Neopterin—He (mM⋅mol^−1^ creatinine)	7.07 ± 2.15	5.97 ± 1.90	0.046 *	−0.861	14
IL-6—He (pg·mL^−1^)	4.18 ± 1.50	3.43 ± 1.11	0.126	−0.982	14
NOx—He (µM)	803 ± 428	449 ± 240	0.020 *	1.022	14
N_2_ 1.45 ATA (20 kPa oxygen)					
ROS—N (μmol⋅min^−1^)	0.238 ± 0.008	0.307 ± 0.018	<0.001 ***	−6.085	9
TAC—N (mM)	0.255 ± 0.025	0.225 ± 0.060	0.189	0.121	9
8-iso-PGF2 ⍺ -N (pg·mg^−1^ creatinine)	364.3 ± 80.56	413.1 ± 95.48	0.281	−0.497	9
Neopterin -N (mM⋅mol^−1^creatinine)	7.87 ± 1.93	5.76 ± 1.68	0.071	−0.857	9
IL-6—N (pg·mL^−1^)	3.82 ± 1.51	5.54 ± 1.98	0.012 *	0.567	9
NOx—N (µM)	671 ± 293	493 ± 189	0.124	0.721	9

**Table 5:** VCAM-1 (μg·L^−1^). Data are shown as mean ± SD (n = sample number) for pre- and post-exposure to both inert gasses. Results are given in mean ± SD. (Paired *t*-test: *p* < 0.05.)

Exposure (60 min)	Baseline	Post-120 min of Exposure	*p* Value	Size Effect	*n*
He 1.45 ATA (20 kPa oxygen)	Value	Value	Paired *t*-test	Cohen’s d	
VCAM-1—He (mg·L^−1^)	580 ± 122	624 ± 126	0.402	−0.349	14
N_2_ 1.45 ATA ((20 kPa oxygen)					
VCAM-1—N (mg·L^−1^)	611 ± 116	561 ± 118	0.351	0.428	9

## Data Availability

The data are available upon request from the authors.

## Abbreviations

DCS	Decompression sickness
EVs	Extracellular vesicles
IL-1β	Interleukin-1β
Il-6	Interleukin-6
MPO	Myeloperoxidase
MPs	Blood-borne microparticles
NOx	Nitric oxide metabolites
pGSN	Plasma gelsolin
PO2	Oxygen partial pressure
RAGE	Receptor for advanced glycation end products
ROS	Reactive oxygen species
TAC	Total antioxidant capacity
TMEM-119	Transmembrane protein 119
TLR4	Toll-like receptor 4
TSP	Thrombospondin 1
VCAM-1	Vascular cell adhesion protein 1
8-iso-PGF2α	8-isoprostane
